# Removal of cranial springs after spring-mediated cranioplasty

**DOI:** 10.3171/2021.1.FOCVID20102

**Published:** 2021-04-01

**Authors:** Christopher L. Kalmar, Jordan W. Swanson, Sameer Shakir, Alexander M. Tucker, Benjamin C. Kennedy, Phillip B. Storm, Gregory G. Heuer, Scott P. Bartlett, Jesse A. Taylor, Shih-Shan Lang

**Affiliations:** Divisions of 1 Plastic and Reconstructive Surgery and; 2 Neurosurgery, Children's Hospital of Philadelphia, Pennsylvania

**Keywords:** sagittal, craniosynostosis, springs, removal, technique, video

## Abstract

Cranial spring hardware is generally removed 3 months after placement for spring-mediated cranioplasty. Spring removal is performed as an outpatient procedure under general anesthesia in approximately 15 minutes through the incision locations of the index procedure. Herein, the authors provide a multimedia demonstration of cranial spring hardware removal after spring-mediated cranioplasty for sagittal craniosynostosis.

The video can be found here: https://vimeo.com/511179695

## Transcript

**0:25 Timeline**. Cranial springs are generally removed approximately 3 months after placement for sagittal craniosynostosis, which reflects several weeks of active expansion followed by about 2 months of consolidation.^1–8^

**0:42 Local Anesthesia**. After general endotracheal anesthesia is induced, local anesthesia containing epinephrine is infused in the patient's scalp incision. The patient is generally left in supine position.

**0:52 Incision**. After prep and drape, the previous scalp incisions are opened to the subgaleal plane, with care taken to protect the underlying cranial bone regenerate.

**1:00 Subgaleal Undermining**. Subgaleal undermining is performed over the midportion of the springs.

**1:07 Dissection of Spring Arms**. Dissection of each of the spring arms is performed to its footplate using tenotomy scissors and Obwegeser elevator.

**1:13 Springs Elevated and Cut**. Each spring is elevated off of the regenerate and then cut with heavy wire cutters in the midline.

**1:19 Springs Removed**. Each hemispring is carefully isolated from its surrounding soft tissue. Heavy needle driver is used to grasp each spring near its footplate; then the spring is axially rotated so that the footplate is retracted from its subcranial position and the hemispring is removed. Rarely, a Kerrison or narrow rongeur can be used to remove cranial bone immediately adjacent to the footplate if it does not easily retract.

**1:47 Irrigation**. Once all springs are removed, the subgaleal space is irrigated and hemostasis assured.

**1:55 Closure**. Layered closure of galea with 3-0 Vicryl and skin with 4-0 plain gut is performed. The spring removal procedure can be performed efficiently in approximately 15 minutes.

**2:06 Dressing**. Bacitracin is applied and a head wrap is optional.

## Author Contributions

Primary surgeon: Lang, Swanson, Storm, Taylor. Assistant surgeon: Lang. Editing and drafting the video and abstract: Lang, Kalmar, Swanson, Shakir, Storm, Heuer. Critically revising the work: all authors. Reviewed submitted version of the work: Lang, Kalmar, Shakir, Kennedy, Heuer, Bartlett, Approved the final version of the work on behalf of all authors: Lang. Supervision: Lang, Bartlett. Filming of video: Kalmar.
